# Supplementary material for: A spatio-temporal brain miRNA expression atlas identifies sex-independent age-related microglial driven miR-155-5p increase
Source: Nat Commun. 2025 May 17;16:4588. doi: 10.1038/s41467-025-59860-6 (PMC12085673; doi:10.1038/s41467-025-59860-6)
Supplement: Supplementary file 1 — Supplementary Information [file 41467_2025_59860_MOESM1_ESM.pdf]

A spatio-temporal brain miRNA expression atlas identifies sex-independent age-related microglial driven miR-155-5p increase

Supplementary Information

Annika Engel[&,1], Viktoria Wagner[&,1,2], Oliver Hahn[2,3], Aulden G. Foltz[2], Micaiah Atkins[2], Amila Beganovic[1], Ian H. Guldner[2,4], Nannan Lu[2,4], Aryaman Saksena[2], Ulrike Fischer[5], Nicole Ludwig[5], Eckart Meese[5], Tony Wyss-Coray[2,4,6], Andreas Keller[1,7,*]

[1] Clinical Bioinformatics, Saarland University, 66123, Saarbrücken, Germany.
[2] Department of Neurology and Neurological Sciences, Stanford University, Stanford, CA, 94305, USA.
[3] Calico Life Sciences LLC, San Francisco, CA, USA.
[4] Wu Tsai Neurosciences Institute, Stanford University School of Medicine, Stanford, CA, USA

[5] Department of Human Genetics, Saarland University, 66421 Homburg/Saar, Germany.
[6] The Phil and Penny Knight Initiative for Brain Resilience, Stanford University, Stanford, CA, USA.
[7] Helmholtz Institute for Pharmaceutical Research Saarland, Helmholtz Center for Infection Research, 66123, Saarbrücken, Germany.
[&] These authors contributed equally: Annika Engel, Viktoria Wagner
* Correspondences should be addressed to Andreas Keller (andreas.keller@ccb.uni-saarland.de; +49 681 30268611).

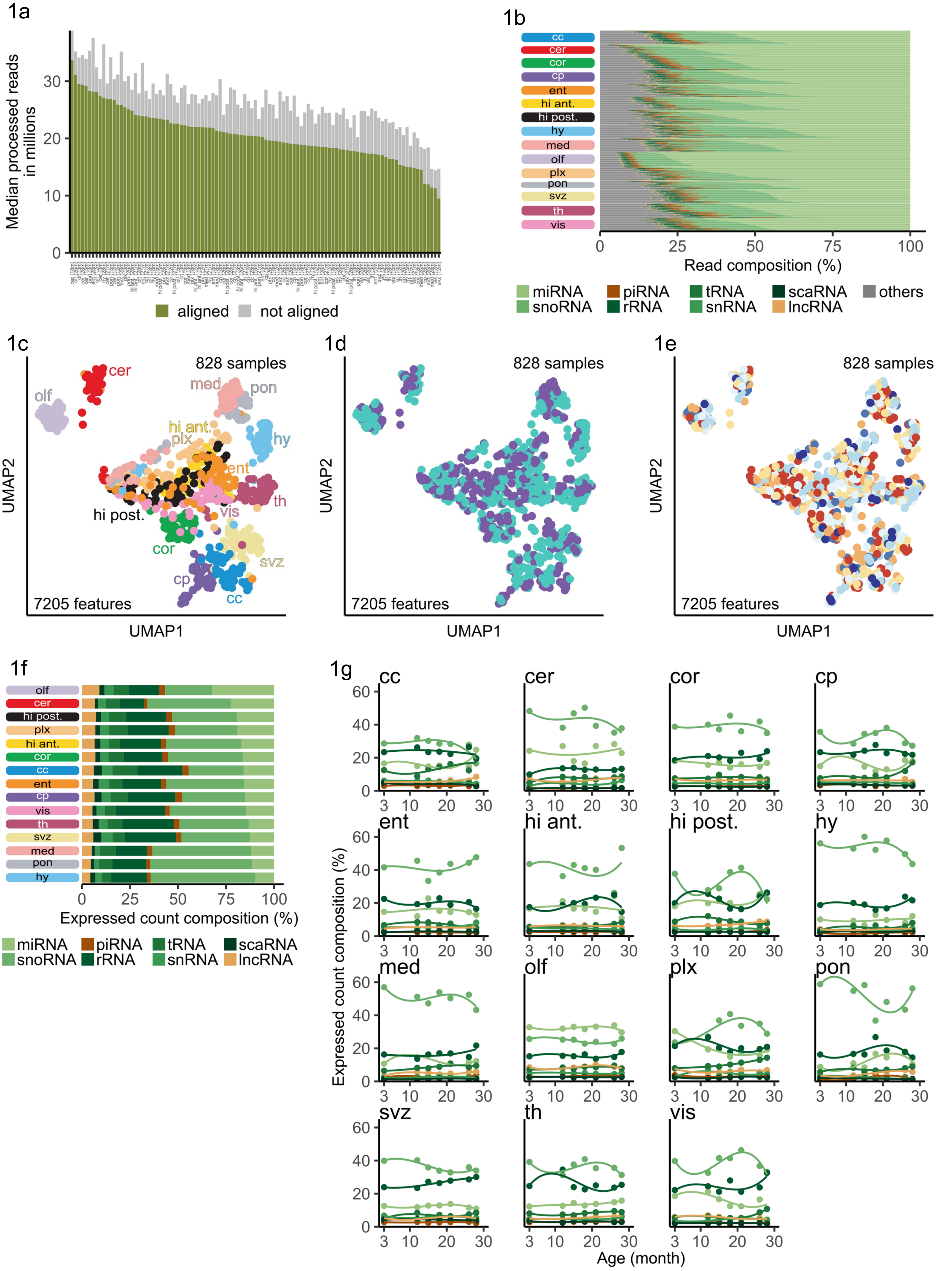

1a
Median processed reads in millions
0
10
20
30
aligned
not aligned
1b
cc
cer
cor
cp
ent
hi ant.
hi post.
hy
med
olf
plx
pon
svz
th
vis
0
25
50
75
100
Read composition (%)
miRNA
piRNA
tRNA
scaRNA
others
snoRNA
rRNA
snRNA
lncRNA
1c
828 samples
UMAP2
UMAP1
7205 features
1d
828 samples
UMAP2
UMAP1
7205 features
1e
828 samples
UMAP2
UMAP1
7205 features
1f
Expressed count composition (%)
1g
Expressed count composition (%)
Age (month)

Supplementary Fig. 1: Overview of read alignment, RNA type distribution, and expression patterns across brain regions, ages, and sexes. **a** Overview of the aligned (green) and not aligned (grey) reads against the mouse genome. Combined per brain region and age. Values are given in millions. **b** Reads mapped to RNA types per sample. Brain regions are highlighted at the left side. **c** UMAP for all regions and all features (miRNAs, lncRNAs, piRNAs, rRNAs, scaRNAs, snoRNAs, snRNAs and tRNAs) colored by brain regions as indicated in Fig. 1a. **d** UMAP of all samples and all features colored by sex. Analogue to Supplementary Fig. 1c. **e** UMAP of all samples and features colored by age. Analogue to Supplementary Fig. 1c. **f** For each brain region and RNA class, we determined the composition of expressed RNA counts, considering only RNAs with raw counts ≥ 5 in at least 10% of all samples from that brain region. **g** For all brain regions and different RNA classes, we analyzed the composition of expressed RNA counts at each age point individually. The trends for all fifteen brain regions are visualized and lines are fitted with a third-degree polynomial**.**

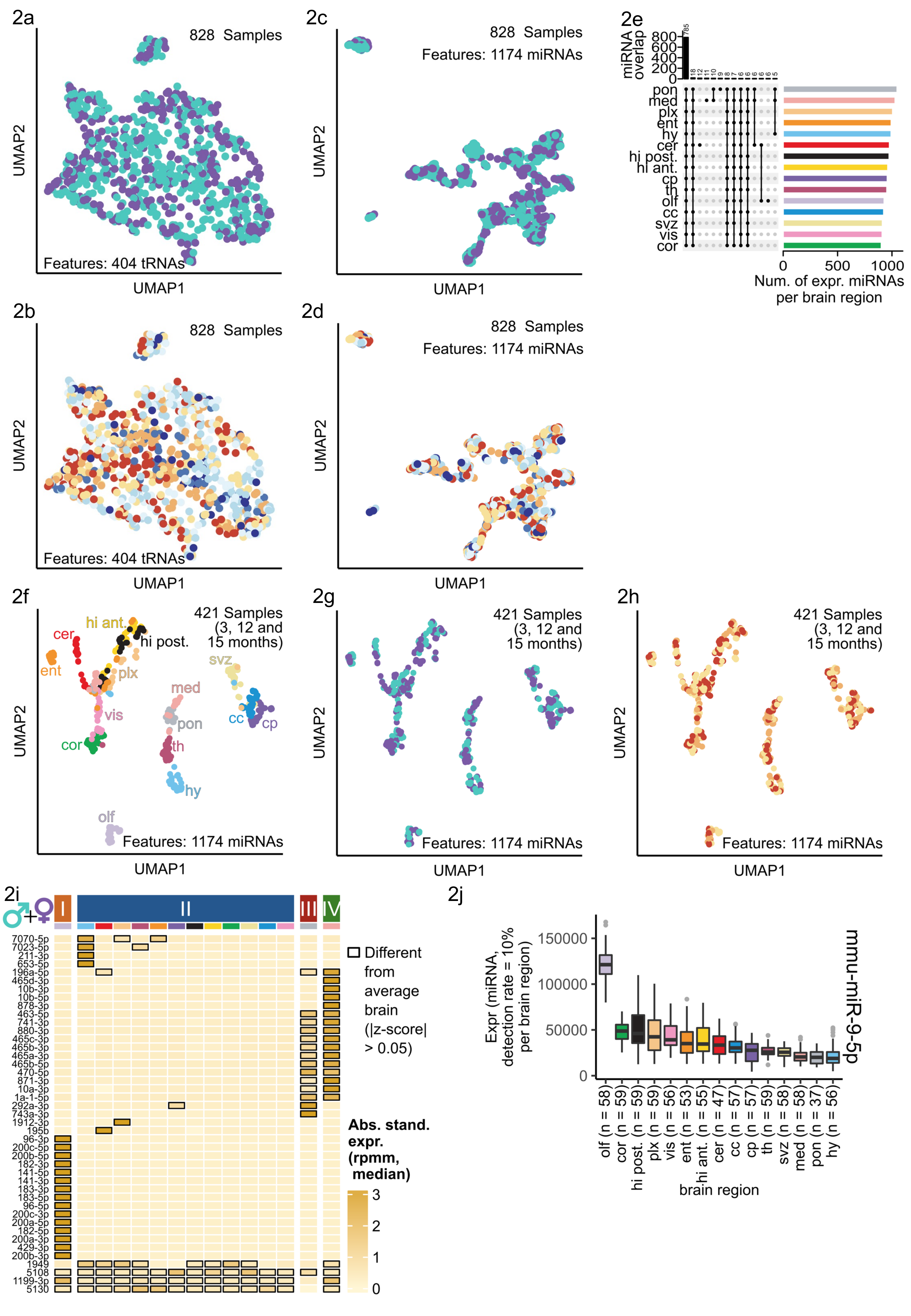
2a
828 Samples
Features: 404 tRNAs
UMAP1
UMAP2
2b
828 Samples
Features: 404 tRNAs
UMAP1
UMAP2
2c
828 Samples
Features: 1174 miRNAs
UMAP1
UMAP2
2d
828 Samples
Features: 1174 miRNAs
UMAP1
UMAP2
2e
miRNA overlap
800
600
400
200
0
785
pon
med
plx
ent
hy
cer
hi post.
hi ant.
cp
th
olf
cc
svz
vis
cor
0
500
1000
Num. of expr. miRNAs per brain region
2f
421 Samples (3, 12 and 15 months)
hi ant.
cer
hi post.
ent
plx
svz
med
vis
pon
cc
cp
cor
th
hy
olf
Features: 1174 miRNAs
UMAP1
UMAP2
2g
421 Samples (3, 12 and 15 months)
Features: 1174 miRNAs
UMAP1
UMAP2
2h
421 Samples (3, 12 and 15 months)
Features: 1174 miRNAs
UMAP1
UMAP2
2i
♂+♀
I
II
III
IV
7070-5p
7023-5p
211-3p
653-5p
196a-5p
465d-3p
10b-3p
10b-5p
878-3p
463-5p
741-3p
880-3p
465c-3p
465b-3p
465a-3p
465b-5p
470-5p
871-3p
10a-3p
1a-1-5p
292a-3p
743a-3p
1912-3p
195b
96-3p
200c-5p
200b-5p
182-3p
141-5p
141-3p
183-3p
183-5p
96-5p
200c-3p
200a-5p
182-5p
200a-3p
429-3p
200b-3p
1949
5108
1199-3p
5130
Different from average brain (|z-score| > 0.05)
Abs. stand. expr. (rpmm, median)
3
2
1
0
2j
mmu-miR-9-5p
Expr (miRNA, detection rate = 10% per brain region)
150000
100000
50000
0
olf (n = 58)
cor (n = 59)
hi post. (n = 59)
plx (n = 59)
vis (n = 56)
ent (n = 53)
hi ant. (n = 55)
cer (n = 47)
cc (n = 57)
cp (n = 57)
th (n = 59)
svz (n = 58)
med (n = 58)
pon (n = 37)
hy (n = 56)
brain region

Supplementary Fig. 2: UMAP of tRNA and miRNA expression across sex, age, and brain regions and clustering analysis of miRNA expressions over the brain regions. **a** UMAP of all samples and for all tRNAs colored by sex. Analogue to Fig. 1d. **b** UMAP of all samples and for all tRNAs colored by age. Analogue to Fig. 1d. **c** UMAP of all samples and for all miRNAs colored by sex. Analogue to Fig. 1f. **d** UMAP of all samples and for all miRNAs colored by age. Analogue to Fig. 1f. **e** Expressed miRNAs per brain region and the overlap between them. **f** UMAP of young samples (3-, 12-, and 15-month-old) colored by brain region. **g** UMAP of all young samples according to Supplementary Fig. 2f colored by sex. **h** UMAP of the young samples according to Supplementary Fig. 2f colored by age. **i** Analogue to Fig. 2a for male and female samples combined: Heatmaps of the 50 top miRNA from all brain regions determined by coefficient of variation calculated using the medians of the expression values of each brain region. Shown are the absolute standardized expression values (z-scores). The black borders are indicating the binarization ($|z\text{-score}| > 0.5$) on which a clustering into four clusters using a hierarchical clustering was performed. For visualization purposes, we removed features entirely below the selected threshold. **j** Overview of the expression values of mmu-miR-9-5p per brain region. The exact number of used samples per each bar are given in the x-axis description. The box plots were generated using the same methodology as described for Fig. 1c and in the Methods section.

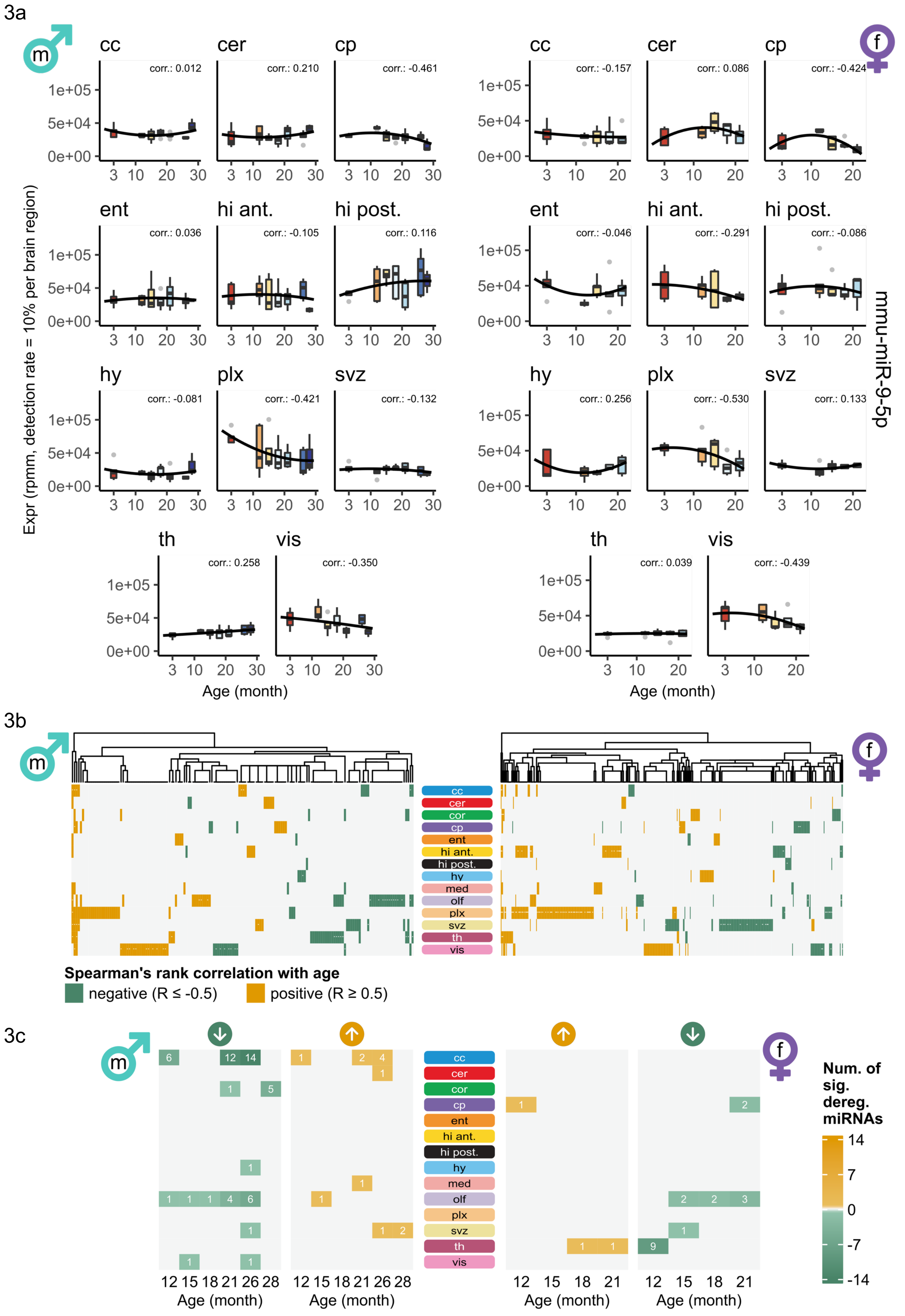
3a
m
f
cc
cer
cp
ent
hi ant.
hi post.
hy
plx
svz
th
vis
corr.: 0.012
corr.: 0.210
corr.: -0.461
corr.: 0.036
corr.: -0.105
corr.: 0.116
corr.: -0.081
corr.: -0.421
corr.: -0.132
corr.: 0.258
corr.: -0.350
corr.: -0.157
corr.: 0.086
corr.: -0.424
corr.: -0.046
corr.: -0.291
corr.: -0.086
corr.: 0.256
corr.: -0.530
corr.: 0.133
corr.: 0.039
corr.: -0.439
1e+05
5e+04
0e+00
3 10 20 30
3 10 20
Expr (rpmm, detection rate = 10% per brain region)
Age (month)
mmu-miR-9-5p
3b
cc
cer
cor
cp
ent
hi ant.
hi post.
hy
med
olf
plx
svz
th
vis
Spearman's rank correlation with age
negative (R ≤ -0.5)
positive (R ≥ 0.5)
3c
12 15 18 21 26 28
12 15 18 21
Age (month)
Num. of sig. dereg. miRNAs
14
7
0
-7
-14

Supplementary Fig. 3: Analysis of age-related miRNA expression changes in mouse brain across sexes. **a** Analog to Fig. 3a. Boxplots showing the other eleven trajectories of miRNA mmu-miR-9-5p. Asterisks highlight the significance of a deregulated comparison between that age and the control age (3 months). The Spearman's rank correlation coefficient from the miRNA with age is displayed above each plot (significantly, if adjusted p-value < 0.05, two-sided Spearman's rank correlation test, Benjamini-Hochberg procedure). The box plots were generated using the same methodology as described for Fig. 1c and in the Methods section, with the exact values provided in Supplementary Data 4 and 5. **b** Spearman's rank correlation coefficient for every miRNA with age per brain region. On the left for male samples and on the right for female samples. Colored areas highlight correlation values above 0.5 (yellow) and underneath -0.5 (green). Asterisks indicate that the corresponding adjusted p-value is significantly (smaller than 0.05, two-sided Spearman's rank correlation test, Benjamini-Hochberg procedure). For clarity of the plot, features were not shown if there was no positive or negative correlation within a brain region. Exact values in Supplementary Data 4. **c** Heatmaps showing for each brain region the number of significantly deregulated miRNAs for the comparison between each older age stage to 3 months (fold change ≥ 1.5 or $\leq 1/1.5$, adjusted p-value < 0.05, two-sided Welch's t-test, Benjamini-Hochberg procedure). Exact values in Supplementary Data 5.

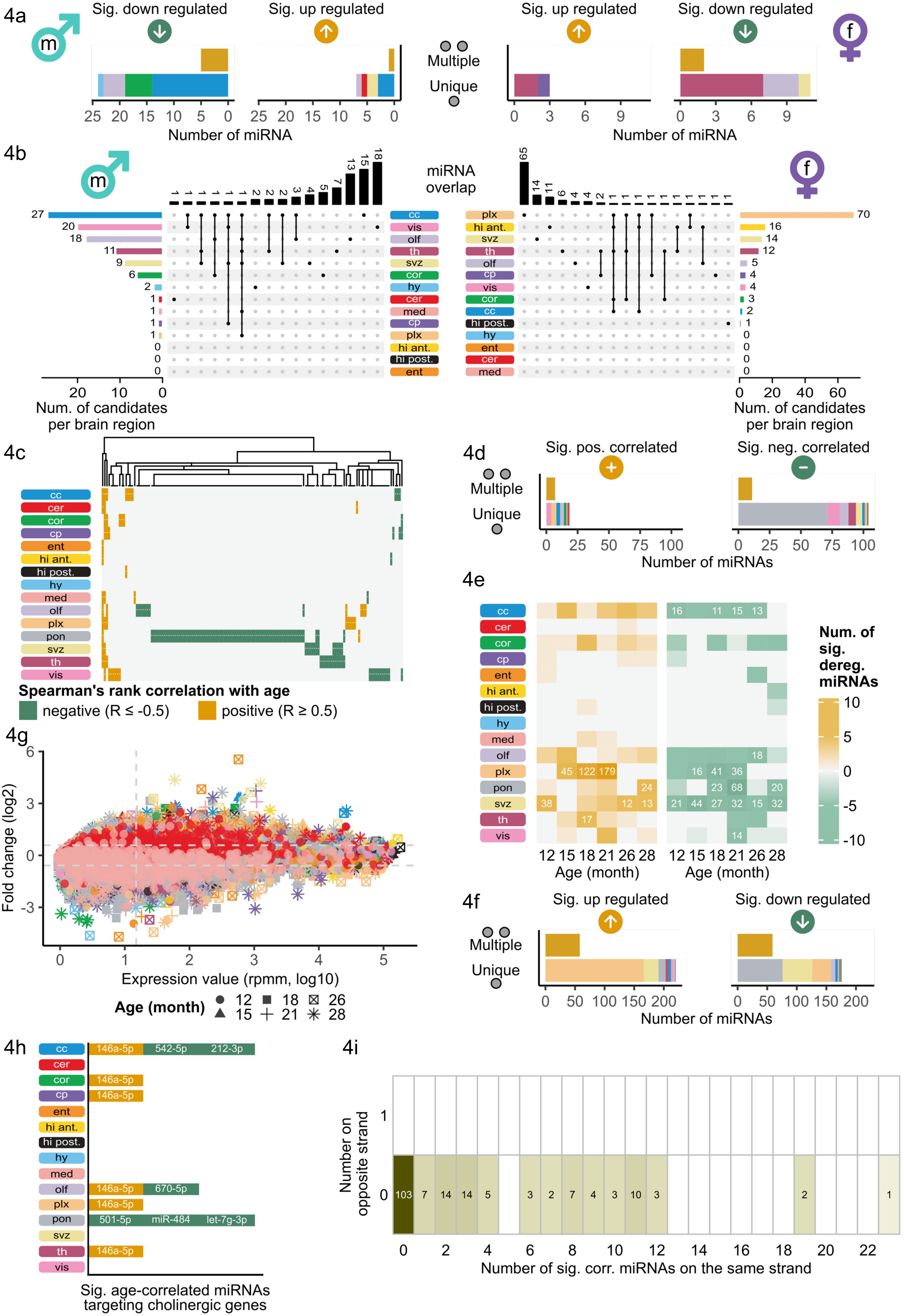

4a
Sig. down regulated
Sig. up regulated
Multiple
Unique
Number of miRNA
4b
miRNA overlap
Num. of candidates per brain region
cc
vis
olf
th
svz
cor
hy
cer
med
cp
plx
hi ant.
hi post.
ent
pon
4c
Spearman's rank correlation with age
negative (R ≤ -0.5)
positive (R ≥ 0.5)
4d
Sig. pos. correlated
Sig. neg. correlated
Number of miRNAs
4e
Age (month)
Num. of sig. dereg. miRNAs
4f
Sig. up regulated
Sig. down regulated
Number of miRNAs
4g
Fold change (log2)
Expression value (rpmm, log10)
Age (month)
12
15
18
21
26
28
4h
146a-5p
542-5p
212-3p
670-5p
501-5p
miR-484
let-7g-3p
Sig. age-correlated miRNAs targeting cholinergic genes
4i
Number on opposite strand
Number of sig. corr. miRNAs on the same strand

Supplementary Fig. 4: Overview of age-associated miRNA expression changes across brain regions, including differential expression, correlation analyses, and genomic neighborhood clustering. **a** Barplots showing significantly deregulated miRNAs for at least one age comparison per region (fold change ≥ 1.5 or ≤ 1/1.5, adjusted p-value < 0.05, two-sided Welch's t-test, Benjamini-Hochberg). Upper bars contain significantly deregulated in multiple regions (yellow). Lower bars contain miRNAs that are unique for one region (colors refer to regions). **b** Upsetplot of miRNAs changing with age per region, showing unique and overlapping candidates from Fig. 3b and Supplementary Fig 4a. **c** Analogous to Supplementary Fig. 3b showing Spearman's rank correlation coefficients for all samples. Colors indicate positive or anti-correlation; asterisks indicate significance (adjusted p-value < 0.05, two-sided Spearman's rank correlation test, Benjamini-Hochberg). Features without correlation to any region are omitted. Exact values in Supplementary Data 7. **d** Analogue to Fig. 3b for all samples combined. Significantly correlated miRNAs with age per region (|R| ≥ 0.5, Spearman's rank correlation coefficient, adjusted p-value < 0.05, two-sided Spearman's rank correlation test, Benjamini-Hochberg). **e** Heatmaps of significantly deregulated miRNAs per region of older ages to 3 months (fold change ≥ 1.5 or ≤ 1/1.5, adjusted p-value < 0.05, two-sided Welch's t-test, Benjamini-Hochberg). Exact values in Supplementary Data 8. **f** Analogous to Supplementary Fig. 4a for all samples combined. Number of miRNAs with at least one significant age comparison (fold change ≥ 1.5 or ≤ 1/1.5, adjusted p-value < 0.05, two-sided Welch's t-test, Benjamini-Hochberg). **g** Scatterplot of log2-fold changes (older ages versus 3 months) against the log10-transformed median expression (excluding 3-month samples). Horizontal lines indicate the fold change thresholds (1.5 and 1/1.5); vertical line at 15 rpmm. **h** Overlap between significantly age-correlated miRNAs (positive yellow and negative green; from Supplementary Fig. 4c) and the predicted miRNAs targeting cholinergic genes[1]. **i** Summary heatmap of significantly age-correlated miRNAs and their neighbors across brain regions. For each region, we counted the neighboring significantly age-correlated miRNAs of each significantly age-correlated miRNA within 10kb on the same and the opposite strand. Overall regions we summarized these occurrences in the heatmap. Heatmap values of 0 are omitted.

5a

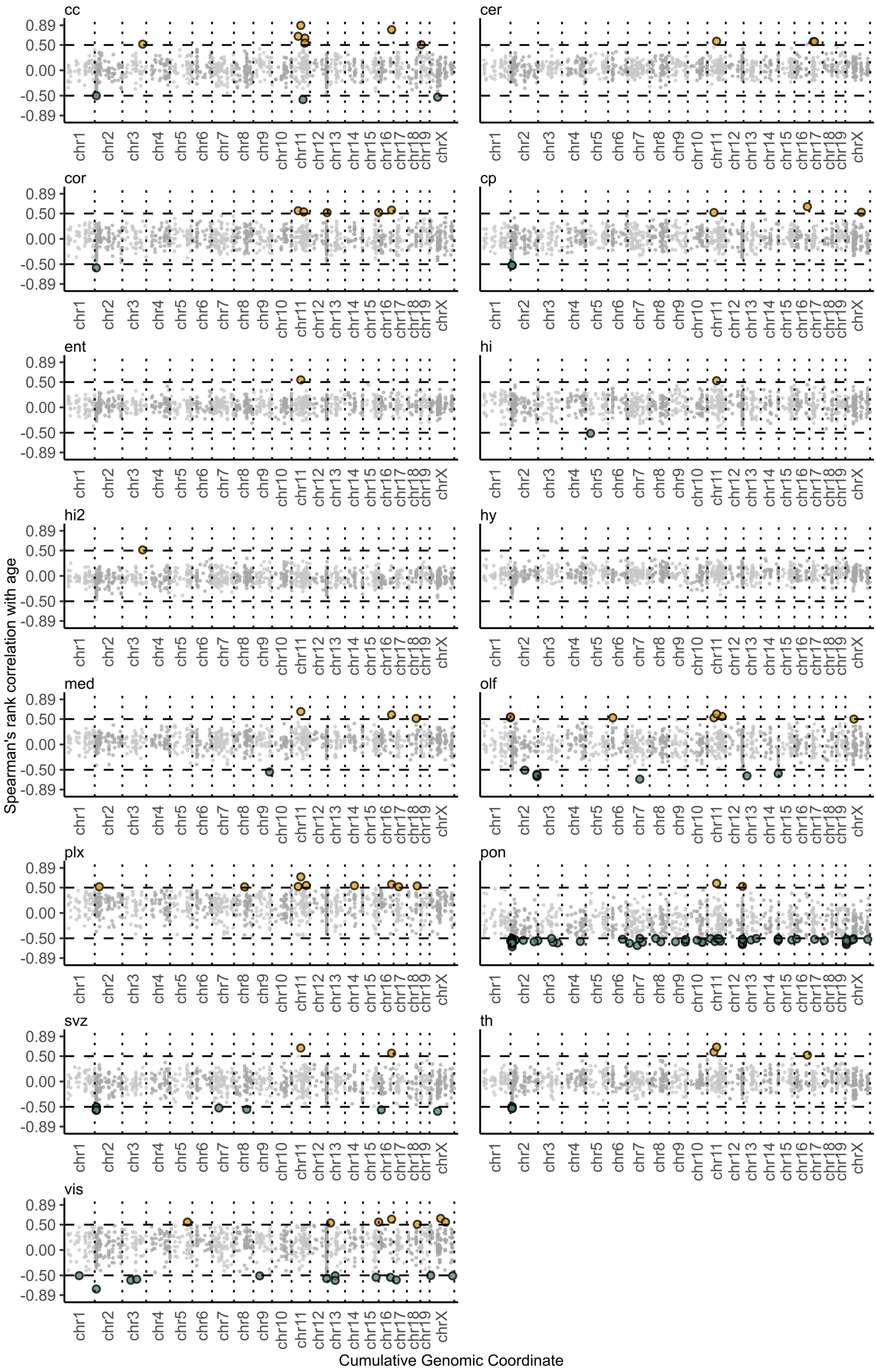
cc
cer
cor
cp
ent
hi
hi2
hy
med
olf
plx
pon
svz
th
vis
0.89
0.50
0.00
-0.50
-0.89
chr1 chr2 chr3 chr4 chr5 chr6 chr7 chr8 chr9 chr10 chr11 chr12 chr13 chr14 chr15 chr16 chr17 chr18 chr19 chrX
Spearman's rank correlation with age
Cumulative Genomic Coordinate

Supplementary Fig. 5: Genomic distribution of age-correlated miRNAs across brain regions. **a** Spearman's rank correlation coefficient values for each miRNA with age per brain region over the cumulative genomic coordinates. Yellow (green) dots indicate significantly positively (negatively) correlated miRNAs (adjusted p-value < 0.05, two-sided Spearman's rank correlation test, Benjamini-Hochberg procedure).

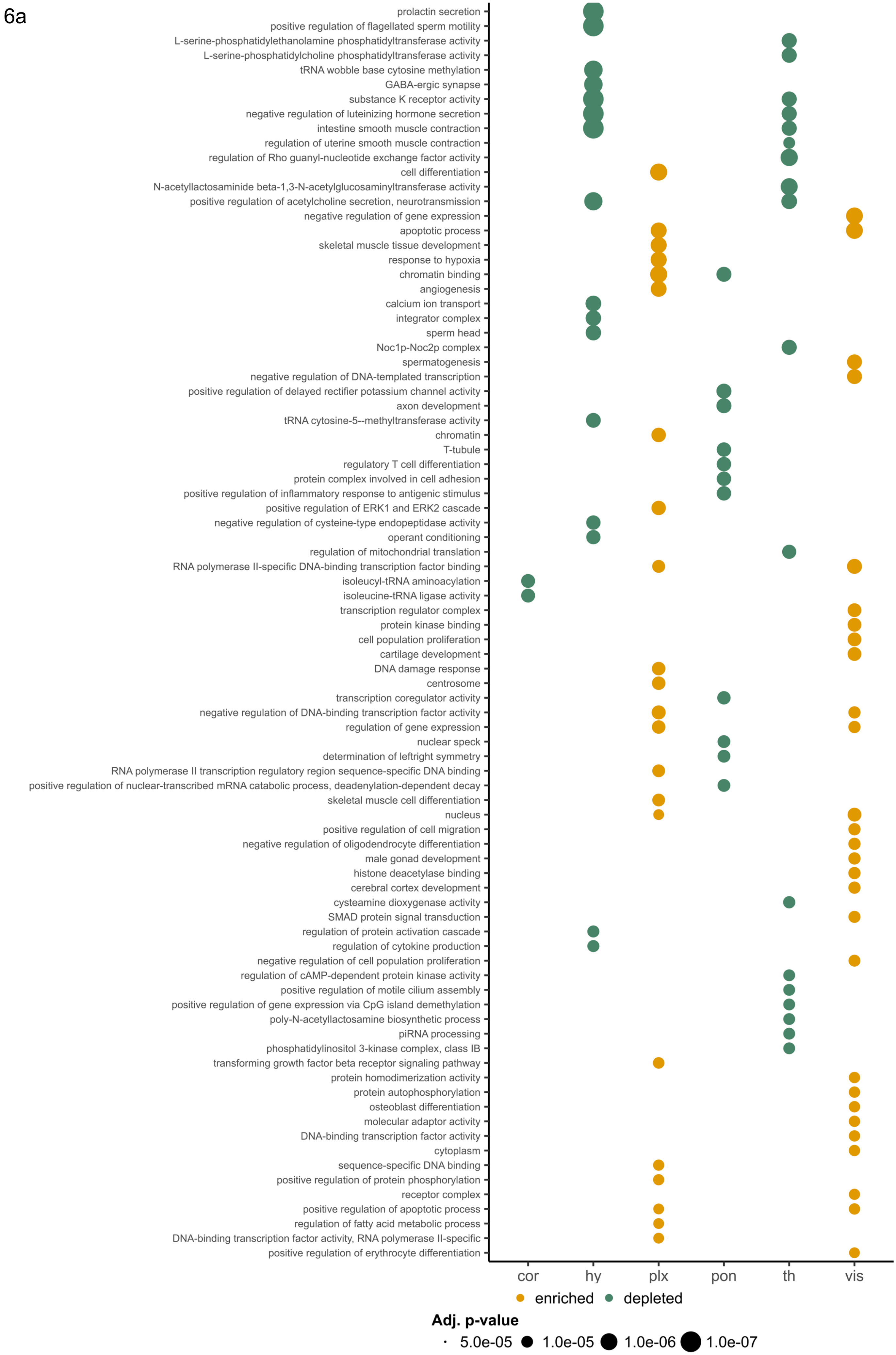
6a
prolactin secretion
positive regulation of flagellated sperm motility
L-serine-phosphatidylethanolamine phosphatidyltransferase activity
L-serine-phosphatidylcholine phosphatidyltransferase activity
tRNA wobble base cytosine methylation
GABA-ergic synapse
substance K receptor activity
negative regulation of luteinizing hormone secretion
intestine smooth muscle contraction
regulation of uterine smooth muscle contraction
regulation of Rho guanyl-nucleotide exchange factor activity
cell differentiation
N-acetyllactosaminide beta-1,3-N-acetylglucosaminyltransferase activity
positive regulation of acetylcholine secretion, neurotransmission
negative regulation of gene expression
apoptotic process
skeletal muscle tissue development
response to hypoxia
chromatin binding
angiogenesis
calcium ion transport
integrator complex
sperm head
Noc1p-Noc2p complex
spermatogenesis
negative regulation of DNA-templated transcription
positive regulation of delayed rectifier potassium channel activity
axon development
tRNA cytosine-5--methyltransferase activity
chromatin
T-tubule
regulatory T cell differentiation
protein complex involved in cell adhesion
positive regulation of inflammatory response to antigenic stimulus
positive regulation of ERK1 and ERK2 cascade
negative regulation of cysteine-type endopeptidase activity
operant conditioning
regulation of mitochondrial translation
RNA polymerase II-specific DNA-binding transcription factor binding
isoleucyl-tRNA aminoacylation
isoleucine-tRNA ligase activity
transcription regulator complex
protein kinase binding
cell population proliferation
cartilage development
DNA damage response
centrosome
transcription coregulator activity
negative regulation of DNA-binding transcription factor activity
regulation of gene expression
nuclear speck
determination of leftright symmetry
RNA polymerase II transcription regulatory region sequence-specific DNA binding
positive regulation of nuclear-transcribed mRNA catabolic process, deadenylation-dependent decay
skeletal muscle cell differentiation
nucleus
positive regulation of cell migration
negative regulation of oligodendrocyte differentiation
male gonad development
histone deacetylase binding
cerebral cortex development
cysteamine dioxygenase activity
SMAD protein signal transduction
regulation of protein activation cascade
regulation of cytokine production
negative regulation of cell population proliferation
regulation of cAMP-dependent protein kinase activity
positive regulation of motile cilium assembly
positive regulation of gene expression via CpG island demethylation
poly-N-acetyllactosamine biosynthetic process
piRNA processing
phosphatidylinositol 3-kinase complex, class IB
transforming growth factor beta receptor signaling pathway
protein homodimerization activity
protein autophosphorylation
osteoblast differentiation
molecular adaptor activity
DNA-binding transcription factor activity
cytoplasm
sequence-specific DNA binding
positive regulation of protein phosphorylation
receptor complex
positive regulation of apoptotic process
regulation of fatty acid metabolic process
DNA-binding transcription factor activity, RNA polymerase II-specific
positive regulation of erythrocyte differentiation
cor
hy
plx
pon
th
vis
enriched
depleted
Adj. p-value
5.0e-05
1.0e-05
1.0e-06
1.0e-07

Supplementary Fig. 6: Gene set enrichment analysis of age-correlated miRNAs across brain regions. **a** The gene set enrichment analysis (GSEA) result created with MIEAA[2] for six brain regions showing the top 50 (sorted by the adjusted p-value, obtained analogously to Fig. 2e) depleted (green) and enriched (yellow) pathways over all brain regions. The analysis was performed for all brain regions individually according to the Spearman's rank correlation coefficient direction and the obtained adjusted p-values (two-sided Spearman's rank correlation test, Benjamini-Hochberg procedure).

7a

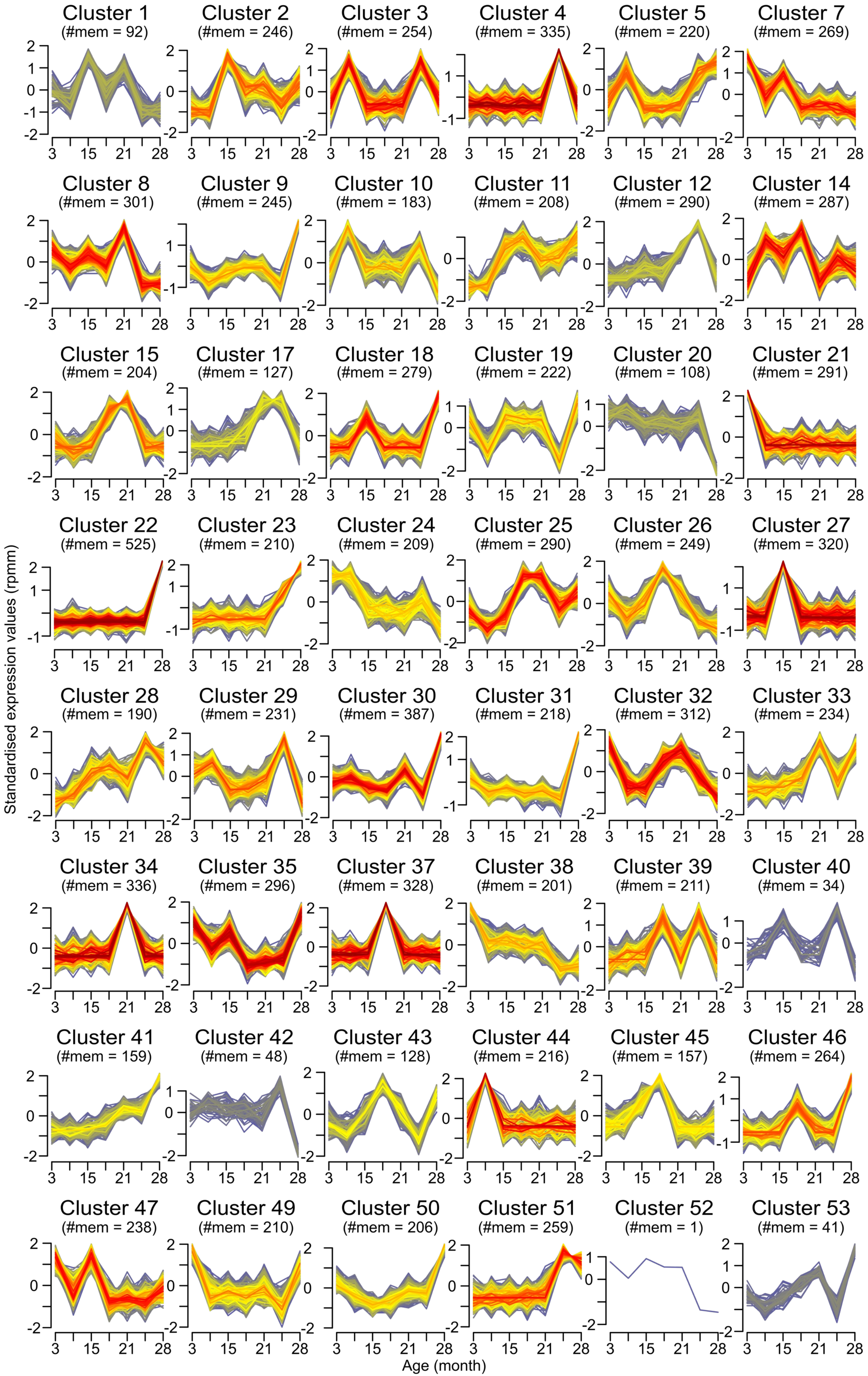
Cluster 1 (#mem = 92)
Cluster 2 (#mem = 246)
Cluster 3 (#mem = 254)
Cluster 4 (#mem = 335)
Cluster 5 (#mem = 220)
Cluster 7 (#mem = 269)
Cluster 8 (#mem = 301)
Cluster 9 (#mem = 245)
Cluster 10 (#mem = 183)
Cluster 11 (#mem = 208)
Cluster 12 (#mem = 290)
Cluster 14 (#mem = 287)
Cluster 15 (#mem = 204)
Cluster 17 (#mem = 127)
Cluster 18 (#mem = 279)
Cluster 19 (#mem = 222)
Cluster 20 (#mem = 108)
Cluster 21 (#mem = 291)
Cluster 22 (#mem = 525)
Cluster 23 (#mem = 210)
Cluster 24 (#mem = 209)
Cluster 25 (#mem = 290)
Cluster 26 (#mem = 249)
Cluster 27 (#mem = 320)
Cluster 28 (#mem = 190)
Cluster 29 (#mem = 231)
Cluster 30 (#mem = 387)
Cluster 31 (#mem = 218)
Cluster 32 (#mem = 312)
Cluster 33 (#mem = 234)
Cluster 34 (#mem = 336)
Cluster 35 (#mem = 296)
Cluster 37 (#mem = 328)
Cluster 38 (#mem = 201)
Cluster 39 (#mem = 211)
Cluster 40 (#mem = 34)
Cluster 41 (#mem = 159)
Cluster 42 (#mem = 48)
Cluster 43 (#mem = 128)
Cluster 44 (#mem = 216)
Cluster 45 (#mem = 157)
Cluster 46 (#mem = 264)
Cluster 47 (#mem = 238)
Cluster 49 (#mem = 210)
Cluster 50 (#mem = 206)
Cluster 51 (#mem = 259)
Cluster 52 (#mem = 1)
Cluster 53 (#mem = 41)
Standardised expression values (rpmm)
Age (month)
3
15
21
28

Supplementary Fig. 7: Clustered expression trajectories of miRNAs per brain region based on c-means clustering. **a** All cluster trajectories with a membership of at least 15% for the c-means cluster result applied to the trajectories consisting of miRNA per brain region. All expression values are standardized and the number of clusters for a membership threshold of 15% are displayed under the cluster number.

8a

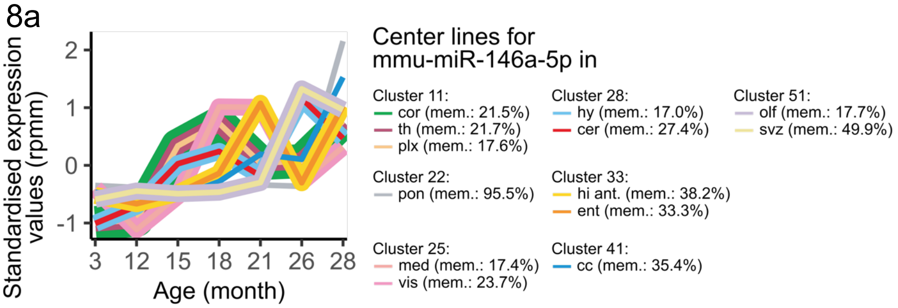

Center lines for mmu-miR-146a-5p in
Cluster 11:
cor (mem.: 21.5%)
th (mem.: 21.7%)
plx (mem.: 17.6%)
Cluster 22:
pon (mem.: 95.5%)
Cluster 25:
med (mem.: 17.4%)
vis (mem.: 23.7%)
Cluster 28:
hy (mem.: 17.0%)
cer (mem.: 27.4%)
Cluster 33:
hi ant. (mem.: 38.2%)
ent (mem.: 33.3%)
Cluster 41:
cc (mem.: 35.4%)
Cluster 51:
olf (mem.: 17.7%)
svz (mem.: 49.9%)
Standardised expression values (rpmm)
Age (month)

8b

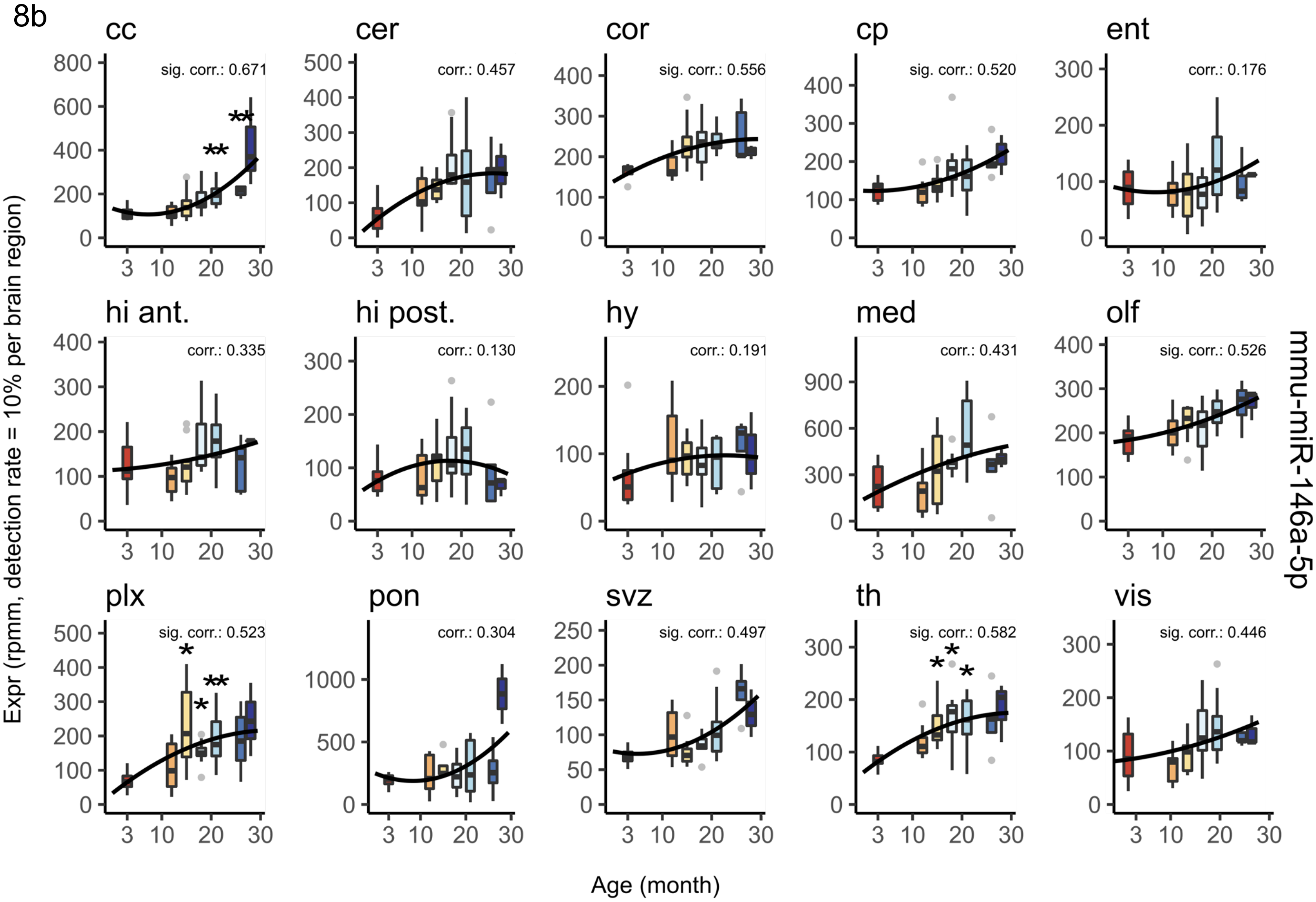

cc
sig. corr.: 0.671
cer
corr.: 0.457
cor
sig. corr.: 0.556
cp
sig. corr.: 0.520
ent
corr.: 0.176
hi ant.
corr.: 0.335
hi post.
corr.: 0.130
hy
corr.: 0.191
med
corr.: 0.431
olf
sig. corr.: 0.526
plx
sig. corr.: 0.523
pon
corr.: 0.304
svz
sig. corr.: 0.497
th
sig. corr.: 0.582
vis
sig. corr.: 0.446
Expr (rpmm, detection rate = 10% per brain region)
mmu-miR-146a-5p
Age (month)

8c

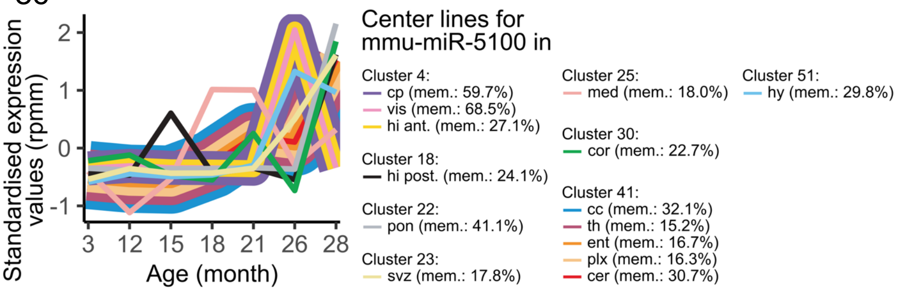

Center lines for mmu-miR-5100 in
Cluster 4:
cp (mem.: 59.7%)
vis (mem.: 68.5%)
hi ant. (mem.: 27.1%)
Cluster 18:
hi post. (mem.: 24.1%)
Cluster 22:
pon (mem.: 41.1%)
Cluster 23:
svz (mem.: 17.8%)
Cluster 25:
med (mem.: 18.0%)
Cluster 30:
cor (mem.: 22.7%)
Cluster 41:
cc (mem.: 32.1%)
th (mem.: 15.2%)
ent (mem.: 16.7%)
plx (mem.: 16.3%)
cer (mem.: 30.7%)
Cluster 51:
hy (mem.: 29.8%)
Standardised expression values (rpmm)
Age (month)

8d

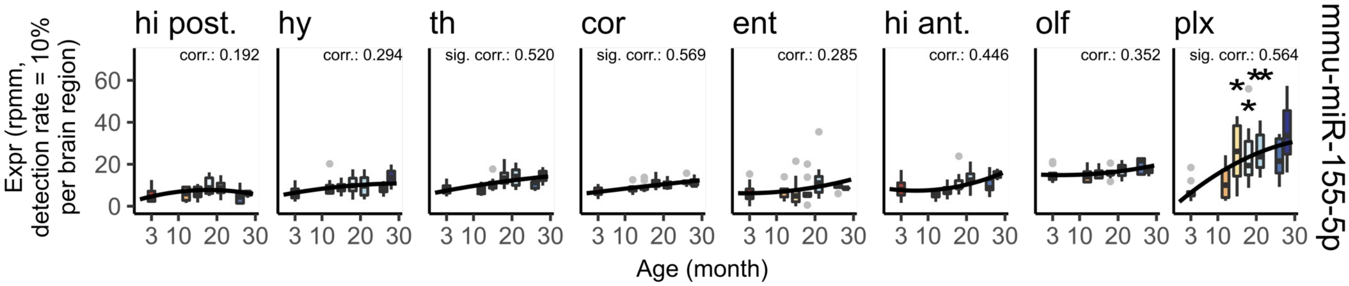

hi post.
corr.: 0.192
hy
corr.: 0.294
th
sig. corr.: 0.520
cor
sig. corr.: 0.569
ent
corr.: 0.285
hi ant.
corr.: 0.446
olf
corr.: 0.352
plx
sig. corr.: 0.564
Expr (rpmm, detection rate = 10% per brain region)
mmu-miR-155-5p
Age (month)

Supplementary Fig. 8: Expression trajectories and cluster assignments of age-related miRNAs across brain regions. **a** The center lines of seven clusters containing trajectories corresponding to mmu-miR-146a-5p. If trajectories for multiple brain regions were assigned to the same cluster and therefore have the same center line, we vary the line width for visualization purposes. **b** The trajectories of miRNA mmu-miR-146a-5p for all fifteen brain regions are provided. Asterisks highlight the significance of a deregulated comparison between that age and the control age (3 months) (fold change ≥ 1.5 or ≤ 1/1.5, adjusted p-value < 0.05, two-sided Welch's t-test, Benjamini-Hochberg procedure). The Spearman's rank correlation coefficient from the miRNA expression with age is displayed above each plot (significantly, if adjusted p-value < 0.05, two-sided Spearman's rank correlation test, Benjamini-Hochberg procedure). The box plots were generated using the same methodology as described for Fig. 1c and in the Methods section, with the exact values provided in Supplementary Data 7 and 8. **c** Analogue to Supplementary Fig. 8a. The center lines of eight clusters containing trajectories corresponding to mmu-miR-5100. **d** Complementing and analogue to Fig. 4h, we see the trajectories from the further eight brain regions of miRNA mmu-miR-155-5p. Asterisks highlight the significance of a deregulated comparison between that age and the control age (3 months) (fold change ≥ 1.5 or ≤ 1/1.5, adjusted p-value < 0.05, two-sided Welch's t-test, Benjamini-Hochberg procedure). The Spearman's rank correlation coefficient from the miRNA expression with age is displayed above each plot (significantly, if adjusted p-value < 0.05, two-sided Spearman's rank correlation test, Benjamini-Hochberg procedure). The box plots were generated using the same methodology as described for Fig. 1c and in the Methods section, with the exact values provided in Supplementary Data 7 and 8.

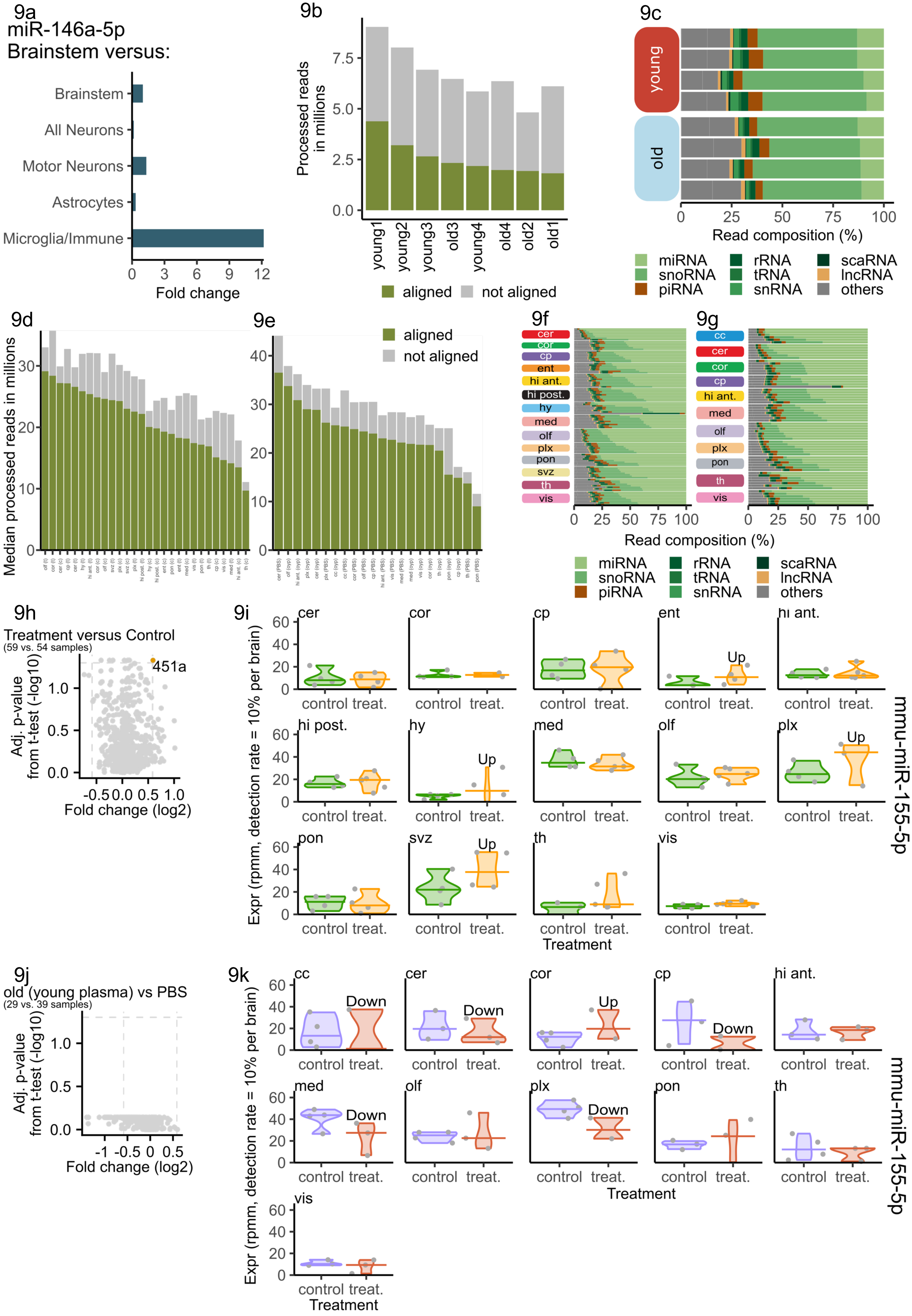
9a
miR-146a-5p
Brainstem versus:
Brainstem
All Neurons
Motor Neurons
Astrocytes
Microglia/Immune
0 3 6 9 12
Fold change
9b
Processed reads in millions
0.0 2.5 5.0 7.5
young1 young2 young3 old3 young4 old4 old2 old1
aligned
not aligned
9c
young
old
0 25 50 75 100
Read composition (%)
miRNA rRNA scaRNA
snoRNA tRNA lncRNA
piRNA snRNA others
9d
Median processed reads in millions
0 10 20 30
9e
aligned
not aligned
0 10 20 30 40
9f
cer cor cp ent hi ant. hi post. hy med olf plx pon svz th vis
9g
cc cer cor cp hi ant. med olf plx pon th vis
0 25 50 75 100
Read composition (%)
miRNA rRNA scaRNA
snoRNA tRNA lncRNA
piRNA snRNA others
9h
Treatment versus Control
(59 vs. 54 samples)
451a
Adj. p-value from t-test (-log10)
0.0 0.5 1.0
-0.5 0.0 0.5 1.0
Fold change (log2)
9i
cer cor cp ent hi ant.
hi post. hy med olf plx
pon svz th vis
Up
control treat.
Expr (rpmm, detection rate = 10% per brain)
Treatment
mmu-miR-155-5p
9j
old (young plasma) vs PBS
(29 vs. 39 samples)
Adj. p-value from t-test (-log10)
0.0 0.5 1.0
-1 -0.5 0 0.5
Fold change (log2)
9k
cc cer cor cp hi ant.
med olf plx pon th
vis
Up
Down
control treat.
Expr (rpmm, detection rate = 10% per brain)
Treatment
mmu-miR-155-5p

Supplementary Fig. 9: Expression patterns and regulation of miRNAs in microglia and under acute dietary restriction (aDR) and young plasma injection (YMP) conditions. **a** Adjusted from CNS microRNA Profiles[3]. Barplot displays fold changes of mmu-miR-146a-5p between brain cell types and brainstem data. **b** Overview of aligned (green) and unaligned (grey) reads against the mouse genome for each sample of the microglia dataset, values in millions. **c** Percentages of reads mapped to RNA types per sample, with ages indicated on the left side. **d** Aligned (green) and unaligned (grey) reads against the mouse genome in the aDR study, grouped by experiment condition, values in millions. **e** Aligned (green) and unaligned (grey) reads against the mouse genome in the YMP study, grouped by experiment condition, values in millions. **f** Percentages of reads mapped to RNA types in the aDR study, brain regions indicated on the left. **g** Percentages of reads mapped to RNA types in the YMP study. **h** Volcano plot of differential expression (treatment versus control) in the aDR study (two-sided Welch's t-test with Benjamini-Hochberg procedure). **i** Violin plots of mmu-miR-155-5p across fourteen brain regions in the aDR study, showing control and treatment (treat.). "Up" indicates upregulated (fold change $\geq$ 1.5) and "Down" downregulated (fold change $\leq$ 1/1.5). Horizontal line mark geometric medians, used for fold change calculation. Exact sample numbers, geometrical medians, fold changes and adjusted p-values in the source data. **j** Volcano plot of differential expression (old mice with young plasma injection (treat.) versus PBS (control)) in the YMP study, analogous to Supplementary Fig. 9h (two-sided Welch's t-test, Benjamini-Hochberg procedure). **k** Violin plots of mmu-miR-155-5p across eleven brains in the YMP study, showing PBS (control) versus old mice with young plasma injection (treat.). As above, "Up" ("Down") indicates upregulation (downregulation) based on fold changes. Horizontal line shows geometric medians used for fold change calculation. Exact sample numbers, geometrical medians, fold changes and adjusted p-values are in the source data.

10a

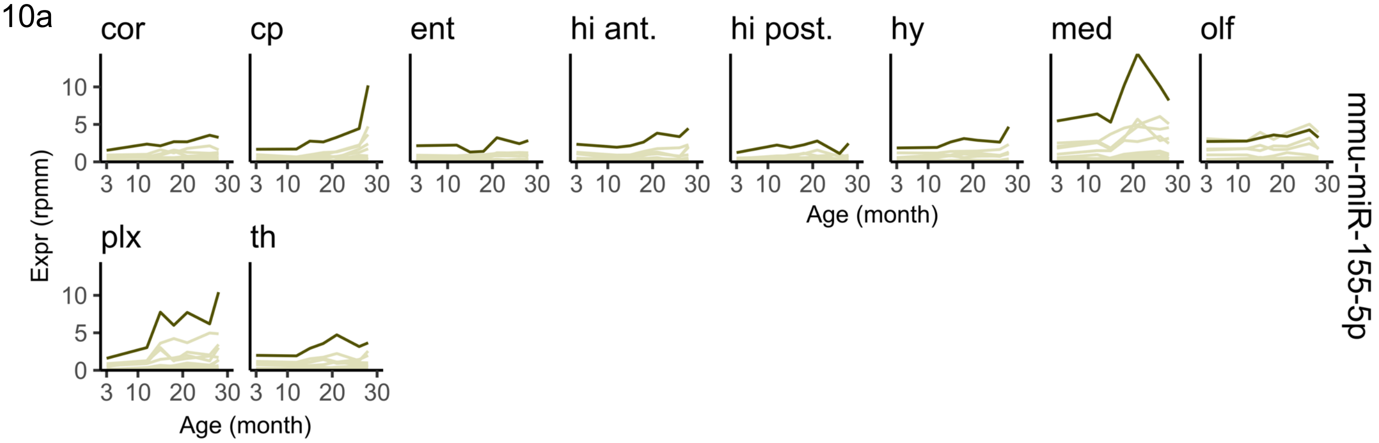

10b

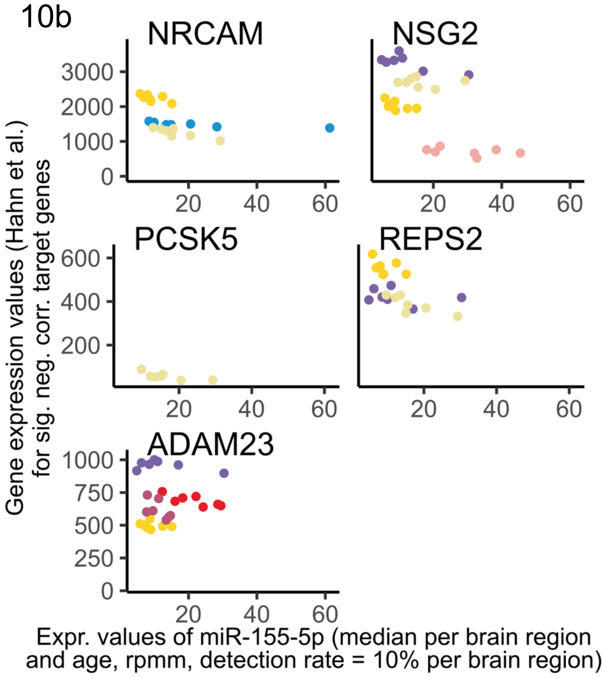

10c

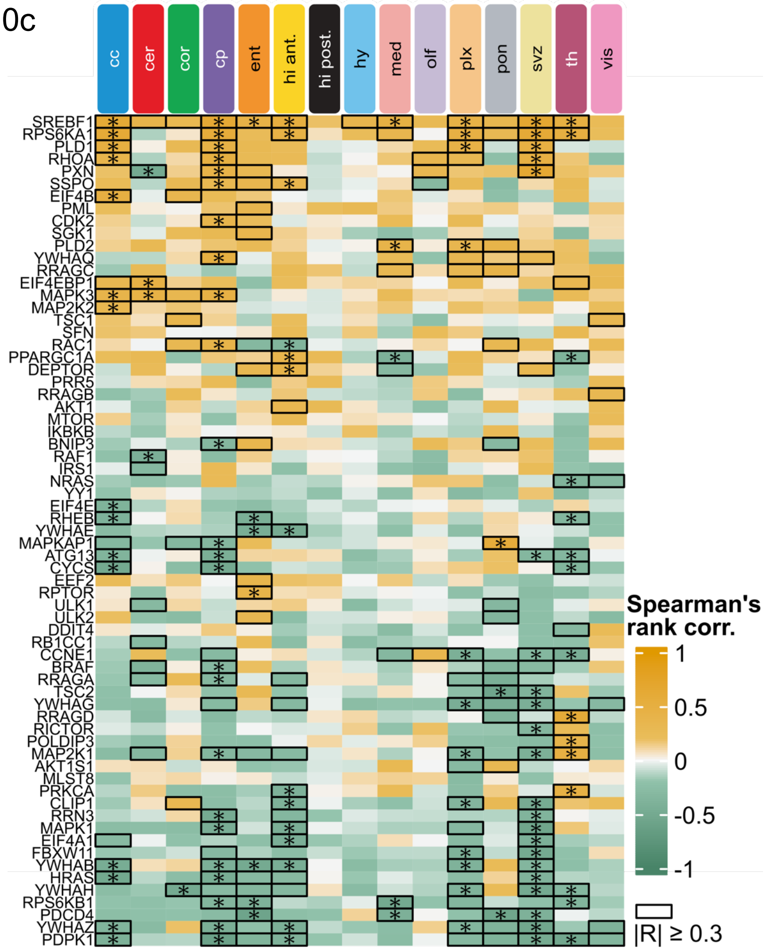

10d

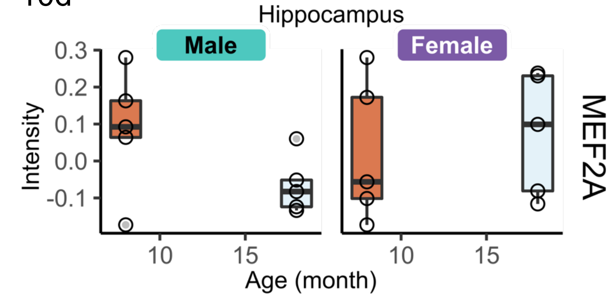

Supplementary Fig. 10: IsomiR expression of mmu-miR-155 and its association with validated target gene expression, mTOR pathway genes, and MEF2A protein levels across brain regions and age. **a** Complementing and analogue to Fig. 5g for the other ten brain regions visualizing the expression of different isomiRs of mmu-miR-155. The canonical isomiR is highlighted as a dark line. **b** The scatter plots are given for five functionally validated target genes[4] with a significant (adjusted p-value < 0.05, two-sided Spearman's rank correlation test, Benjamini-Hochberg procedure) correlation value lower than -0.5. We see in each plot the brain regions in which this gene is significantly anti-correlated (indicated by the color) and the relation between the gene and the miR-155-5p broken down into median per brain region and age point. We used the mRNA data from Hahn et al.[5]. **c** Spearman's rank correlation coefficient values for all genes from the mTOR pathway[6] and mmu-miR-155-5p. Black borders indicate a correlation value below -0.3 or above 0.3 and asterisks denote a significant correlation (p-values obtained with two-sided Spearman's rank correlation test and adjustment by Benjamini-Hochberg procedure). The used mRNA data is from Hahn et al.[5]. **d** Intensity plot of MEF2A in hippocampus split by sex and plotted against the age. Data originates from Aging B6 Proteomics[7]. Black circles mark all data points used for the box plot.

Supplementary References

1 Madrer, N. & Soreq, H. Cholino-ncRNAs modulate sex-specific- and age-related acetylcholine signals. *FEBS Lett* **594**, 2185-2198, doi:10.1002/1873-3468.13789 (2020).

2 Aparicio-Puerta, E. *et al.* miEAA 2023: updates, new functional microRNA sets and improved enrichment visualizations. *Nucleic Acids Res* **51**, W319-W325, doi:10.1093/nar/gkad392 (2023).

3 Pomper, N., Liu, Y., Hoye, M. L., Dougherty, J. D. & Miller, T. M. CNS microRNA profiles: a database for cell type enriched microRNA expression across the mouse central nervous system. *Sci Rep* **10**, 4921, doi:10.1038/s41598-020-61307-5 (2020).

4 Hart, M. *et al.* Expanding the immune-related targetome of miR-155-5p by integrating time-resolved RNA patterns into miRNA target prediction. *RNA Biol* **22**, 1-9, doi:10.1080/15476286.2025.2449775 (2025).

5 Hahn, O. *et al.* Atlas of the aging mouse brain reveals white matter as vulnerable foci. *Cell* **186**, 4117-4133 e4122, doi:10.1016/j.cell.2023.07.027 (2023).

6 Schaefer, C. F. *et al.* PID: the Pathway Interaction Database. *Nucleic Acids Res* **37**, D674-679, doi:10.1093/nar/gkn653 (2009).

7 Keele, G. R. *et al.* Global and tissue-specific aging effects on murine proteomes. *Cell Rep* **42**, 112715, doi:10.1016/j.celrep.2023.112715 (2023).
