# Supplementary material for: A spatio-temporal brain miRNA expression atlas identifies sex-independent age-related microglial driven miR-155-5p increase
Source: Nat Commun. 2025 May 17;16:4588. doi: 10.1038/s41467-025-59860-6 (PMC12085673; doi:10.1038/s41467-025-59860-6)
Supplement: Supplementary file 2 — Description of Additional Supplementary Files [file 41467_2025_59860_MOESM2_ESM.pdf]

Description of Additional Supplementary Files

File Name: supplementary_data_1.xlsx
Description: Metadata table for brain aging, acute diet restriction and young plasma injection studies and the microglia dataset with all sample information containing additional alignment statistics.

File Name: supplementary_data_2.xlsx
Description: Group sizes for brain aging, acute diet restriction and young plasma injection study and the microglia datasets and parameters of the metadata.

File Name: supplementary_data_3.xlsx
Description: Listing the miRNAs included in Fig. 2b per brain region in only male, only female or both.

File Name: supplementary_data_4.xlsx
Description: Spearman's rank correlation coefficient values and the adjusted p-values using the Benjamini-Hochberg procedure for every feature with age for male and female datasets of the aging cohort.

File Name: supplementary_data_5.xlsx
Description: DE analysis results for the corresponding comparisons between the ages and per brain region. Contained are the group sizes, geometric log2-transformed medians, the adjusted p-values (two-sided Welch's t-test) using the Benjamini-Hochberg procedure and the fold changes for male and female datasets of the aging cohort.

File Name: supplementary_data_6.xlsx
Description: A list of all age-related miRNAs obtained by male or female correlation and DE analyses.

File Name: supplementary_data_7.xlsx
Description: Spearman's rank correlation coefficient values and the adjusted p-values using the Benjamini-Hochberg procedure for every feature with age for all samples of aging cohort.

File Name: supplementary_data_8.xlsx
Description: DE analysis results for the corresponding comparisons between the ages and per brain region. Contained are the group sizes, geometric log2-transformed medians, the adjusted p-values (two-sided Welch's t-test) using the Benjamini-Hochberg procedure and the fold for all samples of the aging cohort.

File Name: supplementary_data_9.xlsx

Description: A list of all age-related miRNAs obtained by correlation and DE analyses of the complete aging cohort.

File Name: supplementary_data_10.xlsx

Description: Result of the c-means clustering. Including for every trajectory composed of miRNA and brain region the corresponding cluster and the membership percentage.

File Name: supplementary_data_11.xlsx

Description: DE analysis of the microglia data for the comparison old versus young. Contained are the group sizes, geometric log2-transformed medians, the raw and adjusted p-values (two-sided Welch's t-test) using the Benjamini-Hochberg procedure and the fold for all samples of the microglia.
